# Spiritual beliefs and preferences among patients with multiple myeloma

**DOI:** 10.1007/s00520-026-11444-w

**Published:** 2026-11-09

**Authors:** Danielle Cain, Michelle Ware-Ivy, Benjamin A. Derman, Hannah Johnston

**Affiliations:** 1 Charles R. Drew University of Medicine and Science, Los Angeles, CA USA; 2 The Malcom Orlando Ivy Foundation, Wheaton, IL USA; 3 Section of Hematology and Oncology, Department of Medicine, University of Chicago, Chicago, IL USA

**Keywords:** Religion, Spirituality, Cancer

## Abstract

**Purpose:**

Religion and spirituality are important dimensions of coping and supportive care for many patients with cancer, but their role in the longitudinal care of patients with multiple myeloma is less well characterized. We surveyed patients with multiple myeloma to characterize spirituality and religiosity, examine preferences for incorporating spiritual considerations into care, assess whether spiritual beliefs influence treatment decisions, and explore associations between spirituality or religiosity and myeloma-specific quality of life.

**Patients and methods:**

We developed an anonymous electronic survey incorporating 47 items from three validated survey instruments: the Centrality of Religiosity Scale (CRS), the Functional Assessment of Chronic Illness Therapy–Spiritual Well-Being Scale (FACIT-Sp12), and the EORTC QLQ-MY20, together with investigator-developed questions regarding spiritual beliefs, treatment experiences, and care preferences. Using convenience sampling, surveys were distributed via X/Twitter and within the University of Chicago myeloma patient population. Survey scores were calculated using validated scoring criteria. Group comparisons were performed with the Mann–Whitney *U* test, and associations between spirituality measures and QLQ-MY20 subscales were examined with Spearman’s rank correlation.

**Results:**

Of 281 respondents, 176 (63%) completed all survey components. Median age was 65 years; 64% were female and 85% were non-Hispanic White (NHW; 8.5% non-Hispanic Black, NHB). Most identified as Christian (70%), followed by atheist/agnostic (11%) and spiritual but not religious (10%). Overall, 80% believed clinicians should consider religious or spiritual factors when presenting treatment options and 42% wanted spiritual care services or resources, yet only 19% had discussed spirituality with their care team. Median FACIT-Sp12 and CRS scores were 33 and 3.73, respectively. NHB respondents had higher FACIT-Sp12 and CRS scores indicating higher spiritual well-being, whereas atheist/agnostic participants had lower scores and worse body image. Correlations between spirituality measures and QLQ-MY20 subscales were generally weak.

**Conclusion:**

Spirituality and religiosity vary widely among patients with multiple myeloma, with meaningful differences by race and belief group. Many patients wanted clinicians to consider relevant spiritual or religious factors during treatment discussions, whereas relatively few reported having discussed these beliefs with their oncology team. These descriptive, cross-sectional findings support future study and suggest opportunities to explore how individual spiritual care preferences might be identified and, when desired, met through referral to appropriate resources.

## Introduction

Multiple myeloma is a plasma cell malignancy characterized by clonal proliferation of plasma cells that can lead to anemia, renal dysfunction, hypercalcemia, bone disease, and immune dysfunction, among other complications [1, 2]. More than 36,000 new cases were estimated in the USA in 2025 [3]. Although therapeutic advances have substantially improved outcomes, multiple myeloma generally remains incurable and is often experienced as a chronic, relapsing illness requiring repeated treatment decisions across the disease course [2]. This prolonged disease trajectory, together with treatment-related uncertainty regarding relapse, can impose substantial physical and psychosocial burdens on patients [4].

Religion and spirituality can be critical sources of meaning, coping, and support for patients facing serious illnesses such as malignancy [2, 4, 5]. Prior oncology studies have linked spiritual support and spiritual well-being with quality of life (QOL), coping, and preferences surrounding serious illness care, while also demonstrating that patients and clinicians do not always discuss spiritual concerns despite perceiving them as relevant [6, 7]. Spiritual care has therefore been recognized within palliative and supportive care as one component of multidimensional, patient-centered cancer care [8, 9].

Multiple myeloma provides a distinct context in which to examine these issues as patients face years of sequential therapy and medical decisions regarding clinical trials, cellular therapy, and other intensive treatment [2]. In the limited myeloma-specific literature, religiosity and spirituality have been associated with dimensions of quality of life, but less is known about how patients want spiritual or religious considerations incorporated into routine treatment discussions or whether such beliefs influence acceptance of specific myeloma therapies [10]. Existing cancer literature also suggests that spiritual needs may be addressed by different members of the care team, ranging from basic spiritual inquiry by oncologists to specialist support from chaplains or palliative care teams [4–6].

Accordingly, this study sought to (1) characterize spirituality and religiosity among patients with multiple myeloma; (2) examine preferences regarding consideration of religious or spiritual beliefs during myeloma care and treatment discussions; (3) assess whether spiritual beliefs influence treatment decisions; (4) evaluate variation in these measures across demographic and belief groups; and (5) explore associations between spirituality, religiosity, and myeloma-specific quality of life.

## Methods

A cross-sectional, anonymous electronic survey was developed and administered using the Research Electronic Data Capture (REDCap) system. The questionnaire included 47 items from three validated instruments: the 15-item Centrality of Religiosity Scale (CRS), the 12-item Functional Assessment of Chronic Illness Therapy–Spiritual Well-Being (FACIT-Sp12), and the 20-item European Organisation for Research and Treatment of Cancer Myeloma Module Quality of Life Questionnaire (QLQ-MY20) [11–13]. Investigator-developed items assessed demographics, myeloma treatment history, religious or spiritual affiliation and practices, the perceived influence of spiritual beliefs on treatment decisions, prior discussion of spirituality with the oncology team, and preferences for spiritual consideration or resources in care. Several follow-up items were conditionally displayed on the basis of prior responses; the full survey was estimated to require approximately 10 min to complete.

The CRS assesses the centrality and importance of religious meanings in an individual’s life, where higher scores indicate that religion or spirituality is more integrated into identity and daily life [11]. The FACIT-Sp12 measures spiritual well-being in individuals with chronic illness, with higher scores indicating stronger spiritual well-being [12]. The QLQ-MY20 is a disease-specific QOL instrument for patients with multiple myeloma; for this instrument, higher scores on the disease symptoms and side effects of treatment subscales reflect greater burden, whereas higher scores on the future perspective and body image subscales reflect better functioning and perception [13]. A patient advocate reviewed the investigator-developed survey questions for clarity, cultural appropriateness, and relevance to the lived experience of patients with multiple myeloma. Free-response prompts were included as an exploratory supplement to the quantitative survey. These responses were reviewed by one study author using an inductive approach to identify recurring concepts and illustrative themes. Because the free-text component was not designed as a stand-alone qualitative study, independent dual coding, a formal codebook, assessment of intercoder agreement, and structured coder-reflexivity procedures were not performed.

Eligible participants were adults who self-reported a diagnosis of multiple myeloma. Participants were recruited using convenience sampling through two complementary channels: public distribution of the survey link via X/Twitter, which allowed participation beyond a single institution, and electronic distribution to patients within the University of Chicago myeloma population. Responses were collected anonymously; participants were assigned a unique identifier, and no names, IP addresses, or other direct identifiers were collected. The University of Chicago institutional review board (IRB) determined that this study met criteria for exemption, and our study was conducted in accordance with the Declaration of Helsinki.

Scores for each instrument were calculated using standard instrument-specific scoring. Analyses were performed among respondents who completed all survey components. Group comparisons were performed using the Mann–Whitney *U* test. Correlations between spirituality measures (CRS and FACIT-Sp12) and QLQ-MY20 subscales were assessed using Spearman’s rank correlation. The exploratory free-text responses were used to provide contextual description and were not subjected to inferential statistical analysis.

## Results

### Participant characteristics

A total of 281 individuals initiated the REDCap survey, of whom 176 (63%) completed all sections and were included in the final analysis. The median age of participants was 65 years (range, 31–89), and 64% identified as female. The majority were married or in a committed partnership (82%). The cohort was predominantly non-Hispanic White (NHW, 85%), followed by NHB (8.5%), Hispanic/Latino (3%), and other racial/ethnic backgrounds (3%). The median time since diagnosis of multiple myeloma was 4 years (range, 0–21).

Most participants had undergone autologous stem cell transplantation (ASCT, 61%), 31% had participated in a clinical trial, and 11% had received chimeric antigen receptor (CAR) T-cell therapy. The most common religious affiliation was Christianity (70%), including Protestant (51%), Catholic (36%), and other Christian denominations (13%). Smaller proportions identified as Jewish (6%), atheist or agnostic (11%), spiritual but not religious (10%), or other (3%) (Table 1). For analysis by belief group, atheist and agnostic respondents were combined into one nontheistic category due to the smaller sample size of each independently, recognizing these entities do not represent equivalent belief systems.

**Table 1:** Patient characteristics and spiritual practices

Patient characteristics	All patients (*n* = 176)
Age in years, median (range)	65 (31–89)
Sex, *n* (%)	
Female Male	113 (64%) 63 (36%)
Marital status, *n* (%)	
Married Committed partnership Single Divorced Widowed Separated	136 (77%) 9 (5%) 10 (6%) 10 (6%) 9 (5%) 2 (1%)
Race/ethnicity, *n* (%)	
Non-Hispanic White (NHW) Non-Hispanic Black/African American (NHB) Hispanic/Latino Other	150 (85%) 15 (9%) 6 (3%) 5 (3%)
Years since diagnosis, median (range)	4 (0–21)
Prior treatments, *n* (%)	
Autologous stem cell transplant (ASCT) Chimeric antigen receptor (CAR) T-cell therapy Clinical trial	107 (61%) 19 (11%) 54 (31%)
Religious affiliation, *n* (%)	
Christianity-Protestant Christianity-Catholic Christianity-Other Judaism Atheism/agnostic Spiritual but no religious affiliation Other	63 (36%) 44 (25%) 16 (9%) 10 (6%) 20 (11%) 18 (10%) 5 (3%)
Consulted spiritual advisor on treatment, *n* (%)	46 (26%)
Received spiritual support from faith community, *n* (%)	96 (55%)
Spiritual concerns about, *n* (%)	
ASCT Clinical trials or experimental drugs CAR T-cell therapy Blood transfusions	5 (3%) 4 (2%) 2 (1%) 1 (< 1%)
Delayed/declined treatment due to spiritual beliefs, *n* (%)	3 (2%)
Discussed spiritual beliefs with care team, *n* (%)	34 (19%)

### Spiritual support and decision-making patterns

Participants reported a range of experiences and behaviors related to spirituality during the course of their treatment. More than half (55%) received spiritual support from their faith community, while 26% had consulted a spiritual advisor regarding medical treatment decisions. Few participants reported spiritual concerns related to specific therapies, including ASCT (3%), clinical trials (2%), CAR T-cell therapy (1%), or blood transfusions (< 1%). Only 2% of respondents reported delaying or declining treatment due to spiritual beliefs. Nineteen percent had discussed their spiritual beliefs with their care team (Table 1).

Approximately 40% of respondents viewed their multiple myeloma diagnosis as spiritually or divinely meaningful. A total of 33% reported a change in their spiritual beliefs since learning of their diagnosis, with most (74%) describing a strengthening of faith characterized by increased attendance at faith-based services, more frequent reading of spiritual texts, and/or more frequent prayer.

### Attitudes toward integrating spirituality into care

**Fig. 1:**
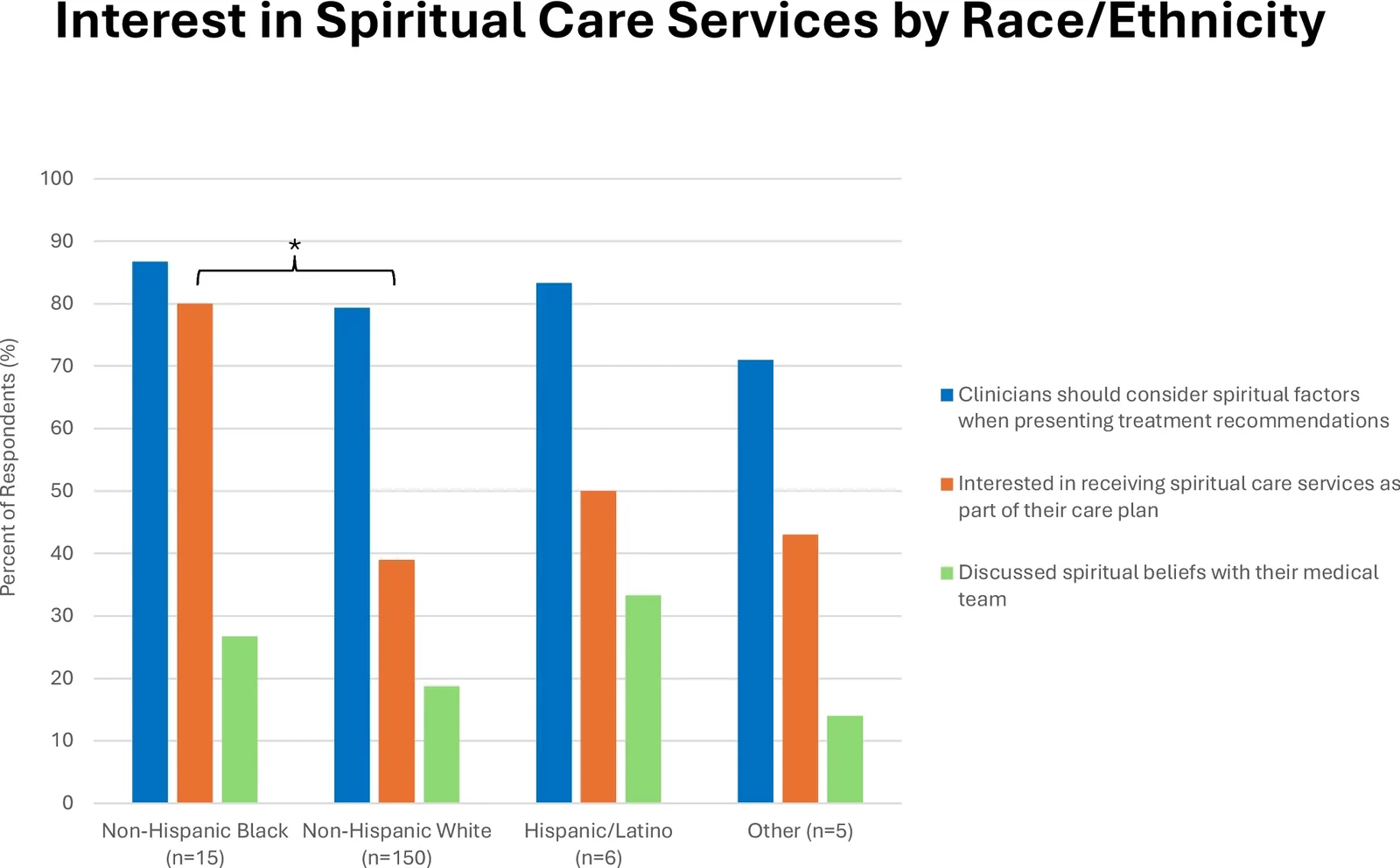
Interest in spiritual care services by race/ethnicity

Most respondents (140/176, 80%) believed that clinicians should consider religious or spiritual factors pertinent to the patient when presenting treatment recommendations. In addition, 75 participants (42%) expressed interest in receiving spiritual care services or inspirational resources as part of their care plan. Despite this, only 19% of respondents had discussed spiritual beliefs with their medical team. Interest in faith-sensitive care was particularly high among NHB respondents, with 80% (12/15) expressing this preference compared with non-NHB respondents (*p* < 0.001) (Fig. 1).

### Spirituality and quality of life measures

**Fig. 2:**
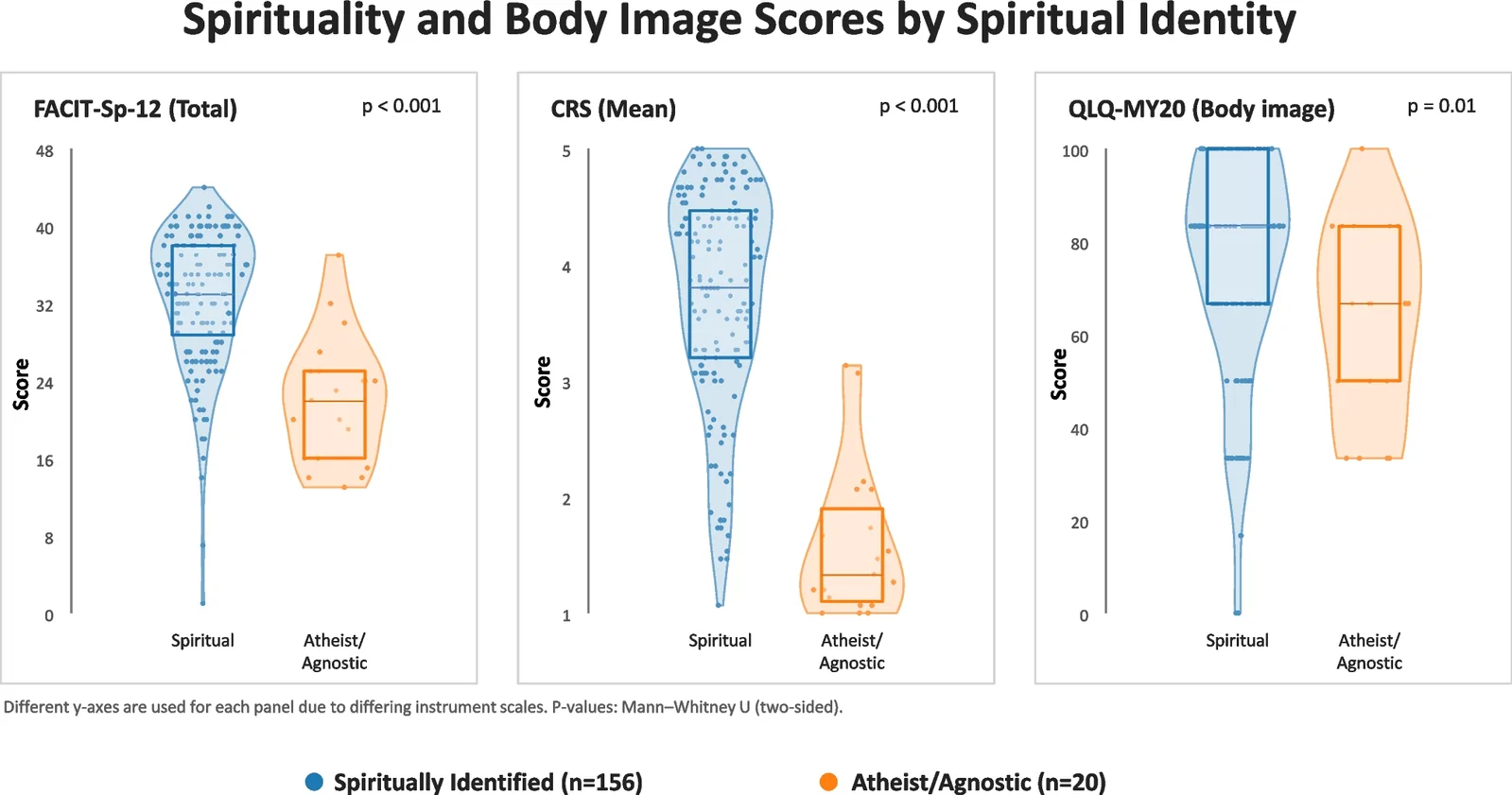
Median spiritual well-being and body image scores among atheist/agnostic vs spiritually identified patients with multiple myeloma. CRS: Centrality of Religiosity Scale; FACIT-Sp12, Functional Assessment of Chronic Illness Therapy–Spiritual; QLQ-MY20, Quality of Life Multiple Myeloma Questionnaire

The median FACIT-Sp12 score for the cohort was 33 (interquartile range [IQR], 26–38), and the median CRS score was 3.73 (IQR, 3.0–4.4) (Table 2). NHB respondents demonstrated significantly higher FACIT-Sp12 (median 37 vs 33, *p* = 0.0085) and CRS scores (4.4 vs 3.67, *p* = 0.0092) than non-NHB respondents (Fig. 2).

**Table 2:** Spirituality and quality of life questionnaire responses

Score	All patients (*n* = 176)	Atheist or agnostic (*n* = 20)	Religious or spiritually identified (*n* = 156)	*p *value
FACIT-Sp12 score^#^				
Total^#^ Meaning/peace Faith	33 21 12	22.5 19 3	35 22 13	< 0.001 0.0067 < 0.001
CRS score^¶^				
Total^¶^ Intellect Ideology Public practice Private practice Religious experience	3.73 3.33 4.67 3 4.67 3.33	1.33 1.33 1.67 1 1.33 1.33	3.87 3.33 5 3.33 4.67 3.87	< 0.001 < 0.001 < 0.001 < 0.001 < 0.001 < 0.001
QLQ-MY20 score, median				
Disease symptoms^*^ Side effects of treatment^*^ Future perspective^^^ Body image^^^	26.67 26.67 66.67 83.33	36.67 46.67 55.56 66.67	26.67 26.67 66.67 83.34	0.54 0.099 0.62 0.01

FACIT-Sp12, Functional Assessment of Chronic Illness Therapy–Spiritual; CRS, Centrality of Religiosity Scale; QLQ-MY20, Quality of Life Multiple Myeloma Questionnaire

^#^Higher scores indicate stronger spiritual well-being

^¶^Higher scores indicate that religion/spirituality is more integrated into their identity and daily life

^*^High scores indicate higher burden

^^^Higher scores indicate improved function

In contrast, atheist/agnostic participants reported significantly lower FACIT-Sp12 (22.5 vs 35, *p* < 0.001) and CRS scores (1.33 vs 3.87, *p* < 0.001) compared with all other respondents. On the QLQ-MY20, atheist/agnostic participants reported lower body image scores (median 66.67 vs 83.34, *p* = 0.01), whereas no significant group differences were observed for disease symptoms, side effects of treatment, or future perspective. Spearman correlation analysis revealed a weak but significant positive correlation between CRS and the disease symptoms subscale (*ρ* = 0.17, *p* < 0.05). No other significant correlations were identified between spirituality measures and QLQ-MY20 subscales.

## Discussion

In this survey of patients living with multiple myeloma, spiritual and religious beliefs were common and heterogenous, but they rarely functioned as a barrier to treatment. Nearly 40% viewed their diagnosis as spiritually or divinely meaningful, and one-third reported strengthened faith since diagnosis. At the same time, only 19% reported discussing spiritual beliefs with their oncology team despite 80% believing clinicians should consider religious or spiritual factors when presenting treatment recommendations. These findings suggest that, for many respondents, spirituality was more closely related to meaning, coping, and support than to refusal of medical therapy. They also identify a potentially important distinction between wanting spiritual beliefs to be acknowledged when relevant and wanting those beliefs to direct treatment decisions.

This pattern is consistent with prior cancer literature in which religion and spirituality have been described as sources of coping, meaning, and support during serious illness [4, 5, 13]. In our cohort, 55% received spiritual support from a faith community, whereas 26% had consulted a spiritual advisor regarding treatment decisions and only 2% reported delaying or declining specific therapies because of spiritual beliefs. Similarly, although 40% interpreted their myeloma as having spiritual or divine meaning, spiritual concerns regarding transplantation, clinical trials, CAR T-cell therapy, and blood transfusions were uncommon. Thus, the clinical relevance of spirituality in this cohort appears to lie less in predicting treatment acceptance or refusal than in understanding how some patients interpret illness and draw on sources of meaning and community during a prolonged disease course.

Our findings regarding patients’ preferences for spiritual consideration should be interpreted within the established oncology and palliative care literature rather than as evidence that spiritual care is absent from cancer practice. Balboni et al. previously demonstrated that religion and spiritual support are important to many patients with advanced cancer and later identified a gap between the perceived importance of spiritual care and its provision by physicians and nurses [4–6]. Consensus recommendations and contemporary ASCO palliative care guidance likewise recognize assessment and support of spiritual needs as components of multidimensional cancer care, acknowledging roles for both oncology clinicians and specialist palliative care or spiritual care providers [7, 8]. Chaplains provide specialist spiritual care in many oncology and palliative care settings, though this may be limited in community practice and in other outpatient settings [14–16]. Against this background, the contribution of our study is disease-specific: 42% of respondents expressed interest in spiritual care services or inspirational resources and 80% wanted clinicians to consider relevant spiritual or religious factors during treatment recommendations, whereas only 19% reported having discussed spiritual beliefs with their oncology team. This preference-experience gap suggests an opportunity to explore how patients with multiple myeloma who want spiritual concerns acknowledged, or who want additional support, might be more consistently identified. Whether such conversations influence the treatments patients receive, trust, alignment between care and patients’ values, or the perceived supportiveness of the clinical relationship cannot be determined from these cross-sectional data and remains a hypothesis for future study. In prior oncology literature, patients’ interest in spiritual consideration appears rooted in its potential to foster trust, validate personal values, and strengthen the patient–physician relationship during challenging care discussions [6].

Our findings also suggest heterogeneity in spiritual experience across racial and belief groups. Enthusiasm for faith-sensitive care was particularly high among NHB respondents, who also reported higher spiritual well-being and religious coping scores. In contrast, atheist/agnostic respondents had markedly lower spiritual well-being and lower body image scores. Racial and ethnic variation in religious coping and spiritual support has been described across multiple oncology and serious illness populations, with NHB patients in particular more frequently reporting religious coping and reliance on spiritual support than NHW patients [17–22]. Our subgroup analyses should nevertheless be interpreted cautiously due to the cross-sectional design and small subgroup size. More broadly, the results reinforce that religiosity, spirituality, and nonbelief are not interchangeable constructs and that spiritual inquiry should be individualized.

This study has several notable strengths. It represents one of the larger investigations focused specifically on spirituality and spiritual care preferences among individuals with multiple myeloma, integrating participants from an academic myeloma program and through an online platform. The use of validated instruments, including the CRS, FACIT-Sp12, and QLQ-MY20, permits assessment of religiosity, spiritual well-being, and myeloma-specific quality of life alongside study-specific questions about treatment decisions and care preferences. The survey was also reviewed by a patient advocate during development to enhance clarity and relevance to the lived experience of patients with multiple myeloma.

Several limitations should be acknowledged. The cross-sectional design precludes causal inference between spirituality, religiosity, and QOL and the study did not assess whether spiritually informed care changes communication, decision quality, treatment adherence, or clinical outcomes. All clinical and treatment information was self-reported and may be subject to recall or social desirability bias. Convenience sampling through a single academic center and X/Twitter could preferentially reach patients who are more technologically literate, engaged with online myeloma communities, or connected to academic care, and the denominator exposed to the survey link could not be determined. The predominance of NHW respondents also limits the generalizability of these findings to more racially and ethnically heterogeneous populations. The free-text component was exploratory and reviewed by a single investigator without independent coding, a formal codebook, or assessment of coder agreement; qualitative interpretations should therefore be considered descriptive rather than the product of a formal qualitative analysis.

In conclusion, this study highlights the prevalence and diversity of spiritual beliefs among patients with multiple myeloma and demonstrates their association with patients’ well-being and care preferences. Spirituality and religiosity were important to many patients with multiple myeloma in this survey, but spiritual beliefs seldom led respondents to delay or decline treatment. The more prominent signal was a preference among many patients for relevant spiritual or religious factors to be acknowledged during care, coupled with relatively infrequent reported discussion with the oncology team.

Existing oncology and palliative care frameworks offer practical models for responding to such preferences: clinicians can use brief, patient-directed inquiry to identify sources of meaning or spiritual concerns, recognize spiritual distress, and offer referral to chaplaincy, palliative care, or a patient’s own faith or spiritual community when desired [7, 8, 17, 23, 24]. This approach does not require oncologists to provide specialist spiritual care, but they should remain cognizant to individual preference, including the preferences of patients who do not identify as religious or spiritual. Future studies in multiple myeloma should prospectively evaluate the acceptability and uptake of such approaches and determine whether systematically identifying and responding to spiritual care preferences affects patient experience, communication, or decision quality.

## Data Availability

Our de-identified data are available upon reasonable request and with the permission of authors.
